# The effect of ICU diary on psychiatric symptoms after ICU discharge among adult critically ill patients: A prospective comparative study

**DOI:** 10.1002/ams2.70026

**Published:** 2024-11-28

**Authors:** Mami Shibata, Kyohei Miyamoto, Nozomu Shima, Tsuyoshi Nakashima, Junichi Fukushima, Shinichi Yamada, Sohei Kimoto, Shigeaki Inoue

**Affiliations:** ^1^ Department of Emergency and Critical Care Medicine Wakayama Medical University Wakayama City Japan; ^2^ Department of Neuropsychiatry Wakayama Medical University Wakayama City Japan

**Keywords:** anxiety, depression, ICU diaries, post‐intensive care syndrome, post‐traumatic stress syndrome

## Abstract

**Aim:**

Psychiatric problems are common in critically ill patients after discharge from an intensive care unit (ICU). The effect of intensive care unit (ICU) diaries on psychiatric symptoms after ICU discharge was investigated in this prospective study.

**Methods:**

Enrolled were critically ill adult patients who were emergently admitted to an ICU and expected to stay for at least 2 days. These patients received ICU diaries filled out by healthcare professionals and family members. Comparison was made with a historical cohort from a previous trial conducted in the same ICU but without ICU diaries. The primary outcome was the presence of significant post‐traumatic stress disorder (PTSD) symptoms 3 months after ICU discharge. Secondary outcomes included significant symptoms of anxiety and depression.

**Results:**

Among 61 patients with ICU diaries, questionnaires were sent to 44 patients 3 months after ICU discharge; 29 patients responded and were analyzed (ICU diary group). Seventy‐four patients from a historical cohort were used as a control group. The proportion of patients with significant PTSD symptoms was 19% in the ICU diary group and 16% in the control group (adjusted odds ratio [aOR] [95% confidence interval: 95% CI]: 0.98 [0.26–3.70]). For anxiety and depression, the proportions were 25% and 29% in the ICU diary group, and 38% and 45% in the control group (aOR [95% CI]: 0.46 [0.15–1.38] for anxiety, aOR [95% CI] 0.40 [0.14–1.16] for depression).

**Conclusion:**

ICU diaries were not associated with a reduced incidence of PTSD symptoms 3 months after ICU discharge.

## Graphical abstract

This prospective study examined the effect of ICU diaries on psychiatric symptoms after ICU discharge in critically ill patients. ICU diaries did not decrease the incidence of PTSD (19% in the ICU diary group and 16% in the control group). Numerically lower incidence of anxiety (25% vs. 29%) and depression (38% vs. 45%) was observed in the ICU diary group.

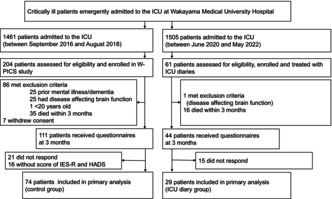

## INTRODUCTION

Post‐intensive care syndrome (PICS), represented by long‐term physical, mental, and cognitive dysfunction after critical illness, is becoming a major concern in modern society.^1^ This is due to the increasing number of patients with critical illness who survive for long periods.^2^ Concerning psychiatric symptoms, approximately half of these patients have post‐traumatic stress disorder, anxiety, or depressive symptoms.^3^, ^4^

The mechanisms underlying psychiatric symptoms in PICS are still unclear, but some studies have suggested that delirium during intensive care unit (ICU) stays and the delusional memories resulting from delirium may contribute to psychiatric symptoms after critical illness.^5^, ^6^ ICU survivors often have delusional memories that do not accurately reflect what occurred in the ICU, and this can subsequently have a negative impact on their long‐term mental health.^5^ ICU diaries have been used to address this, in which the entries are recorded by healthcare professionals and the patient's family members during their ICU stay. This intervention was aimed at preventing long‐term psychiatric symptoms among ICU survivors by filling potential gaps in their memories during their ICU stay and by correcting delusional memories.^7^

A recent systematic review and meta‐analysis reported that the use of ICU diaries led to a reduced incidence of depression symptoms and improvement of health‐related quality of life.^8^ By contrast, a large multicenter randomized trial did not observe a decrease in psychiatric symptoms such as post‐traumatic stress disorder (PTSD), depression, and anxiety.^9^ This discrepancy might be partly explained by the manner in which ICU diaries were implemented, including the incorporation of post‐discharge debrief sessions, the various symptoms explored (e.g., PTSD, anxiety and depression), and the different instruments utilized to evaluate psychiatric symptoms.^10^ Additional investigations are thus suggested to be necessary to clarify the impact of ICU diaries.

This study examined whether the implementation of ICU diaries was associated with a reduction in the prevalence of psychiatric symptoms (including PTSD symptoms) in an academic hospital in Japan.

## MATERIALS AND METHODS

In this single‐center prospective comparative study, a historical cohort (between September 2016 and August 2018) was used as a control. It was conducted in the ICU of Wakayama Medical University Hospital, Wakayama, Japan, between June 2020 and May 2022. The study was approved by the Wakayama Medical University Ethics Boards (approval number 2884) and was conducted in accordance with the Declaration of Helsinki. It was retrospectively registered to the UMIN Clinical Trial Registry on 9th November, 2020 after the initiation of patient enrollment, given the noninvasive nature of our study (registration no. UMIN000042310, https://center6.umin.ac.jp/cgi‐open‐bin/ctr_e/ctr_view.cgi?recptno=R000048303). ICU diaries were used for the patients enrolled in this study and their outcomes were compared with those observed in the historical cohort selected from the Wakayama‐PICS study.^4^ The sample size was set as 40 patients, based on the expected number of patients during the study period and expected response rate for questionnaires.

During the study period, consecutive patients were screened for eligibility and enrolled in this study. The inclusion criteria for this study were as follows: emergent admission to the ICU, age ≥20 years, expected ICU stay ≥48 h, and family members being able to visit and write ICU diaries. Patients were excluded from the study if they were admitted to the ICU for diseases that could directly affect brain function (e.g., brain injury, hypoxemic encephalopathy, stroke, or acute poisoning), had preexisting mental illness including dementia before ICU admission, had no family members who could visit them during their ICU stay, had end‐stage malignancy with a life‐expectancy <1 year, had a decision to withhold or withdraw life‐sustaining treatment, were enrolled in this study during a previous ICU admission, or were otherwise deemed inappropriate for enrollment by attending physicians.

The criteria for selecting the historical cohort from the Wakayama‐PICS study cohort and the evaluation of outcomes in the Wakayama‐PICS study were consistent with the inclusion and exclusion criteria as well as the outcome evaluation in the current study. The Wakayama‐PICS study, conducted as a single‐center, prospective, observational study, enrolled 204 patients aged ≥20 years who were admitted to our ICU between September 2016 and August 2018. The Wakayama‐PICS study aimed to assess activity of daily living (ADL) and psychiatric symptoms such as PTSD, anxiety, and depression at 3 and 12 months after ICU discharge. Anxiety and depression were assessed with Hospital Anxiety and Depression Scale (HADS),^11^ PTSD with Impact of Event Scale‐Revised (IES‐R),^12^ and ADL with Barthel index (BI).^13^ The results of Wakayama‐PICS study have been published elsewhere.^4^, ^6^, ^14^, ^15^, ^16^

Before the initiation of this study, practical and educational writing guidelines were developed to be used by ICU medical staff, including physicians, nurses, and physiotherapists, which detailed how to compose ICU diaries. This instructional material was made available for reference by ICU staff at any time. For the family members, ICU medical staff taught them how to write ICU diaries according to the writing guidelines. Patient enrollment commenced after a preparatory period during which the use of ICU diaries was trialed for several patients (who were not included in this study).

ICU physicians screened eligibility and obtained written informed consent from patients or their family members. On the first day of ICU admission, the attending physicians documented a summary of the diagnosis, present illness, and treatment plan on the initial page of the ICU diaries (Figure S1). Starting from the second day of ICU admission, both ICU physicians and nurses contributed entries to the patient's diary, chronicling the day's treatment and the patient's condition, occasionally accompanied by pictures of the patient. Family members were encouraged to contribute to the diaries during their visits. Notably, however, family visits were infrequent throughout the study period because our hospital restricted family visitation due to the coronavirus pandemic from September 2020 until the end of the study in May 2022.

Upon patients' recovery from critical illness and regaining clear consciousness during hospitalization, the ICU diaries were directly presented to the patients themselves. In cases where patients had not regained consciousness due to delirium or cognitive impairment, the diaries were instead handed to their family members. Whenever possible, debriefing sessions about the patients' ICU stay were conducted with both patients and their family members during the diary handover. If direct handover and debriefing were not feasible, such as in cases where patients or their family members were unavailable, the ICU diaries were mailed to them after hospital discharge.

Three months after ICU discharge, questionnaires were mailed to the patients (mirroring the approach employed in the Wakayama‐PICS study) which encompassed the IES‐R, HADS, and BI assessments. The questionnaires also included inquiries related to the handling of ICU diaries. These supplementary questions delved into aspects such as the frequency with which patients perused their diaries, the perceived utility of the diaries, and the extent to which patients perceived differences between the diary contents and their own memories about their ICU stay. Patients were informed that they had the option to be referred to the specialized psychiatric outpatient department in our hospital if they experienced psychiatric symptoms.

The primary outcome was the presence of significant PTSD symptoms at 3 months after ICU discharge. The primary outcome was assessed at this specific time point because it aligns with the most commonly utilized timing in PICS studies, including those involving ICU diaries.^8^, ^10^ The score of IES‐R ranges from 0 to 88 points, with higher score indicating more severe symptoms, and significant PTSD symptoms were defined as those ≥25 points.^17^ Secondary outcomes included the presence of anxiety, depression, and impairment in basic ADL at 3 months after ICU discharge. Anxiety and depression were evaluated using HADS, which comprises two subscales: HADS‐Anxiety and HADS‐Depression, each scored from 0 to 21 points. A score of ≥8 on either subscale was defined as the presence of significant symptoms.^18^ Basic ADL disability was assessed through BI, rated from 0 to 100, with higher scores indicating greater independence. The BI comprises 10 items: feeding, transfers (bed to chair and back), grooming, toilet use, bathing, mobility, stairs, dressing, bowels, and bladder. A BI score of ≤60 was defined as a disability in basic ADL.^19^

### Statistical analysis

Continuous variables are presented as median and interquartile range (IQR), and categorical variables are presented as numbers and percentages (%). For comparison of the two groups, the Wilcoxon rank‐sum test was used for continuous variables, and Fisher's exact test was used for categorical variables. To evaluate the association between the ICU diary and psychiatric symptoms, univariate and multivariate logistic regression analyses were used. The multivariate model was constructed using predefined adjusters selected based on previous literature and clinical judgment. These adjusters included older age (≥70 years old), sex, disease severity (Acute Physiologic Assessment and Chronic Health Evaluation [APACHE] II score ≥ 20), length of ICU stay (>5 days), and the presence of delirium or coma during the ICU stay.^6^, ^20^ As post hoc analysis, propensity score‐based analysis was constructed as sensitivity analysis. The propensity score for ICU diary use was calculated with a logistic regression model that included all measured variables related to patient characteristics and variables during ICU stay (e.g., age, sex, APACHE II score, SOFA score, disability prior to ICU admission, Charlson comorbidity index, presence of sepsis, postoperative status, vasopressor therapy, mechanical ventilation, kidney replacement therapy, delirium, coma, and ICU stay >5 days). Patients were stratified into five groups based on their propensity scores and these groups were adjusted in a multivariate logistic regression model. There were no missing data regarding these adjusters. A two‐sided *p* value < 0.05 was considered statistically significant, and all analyses were performed using JMP Pro Software (version 14.0.0; SAS Institute Inc., Cary, NC, USA).

## RESULTS

**FIGURE 1:**
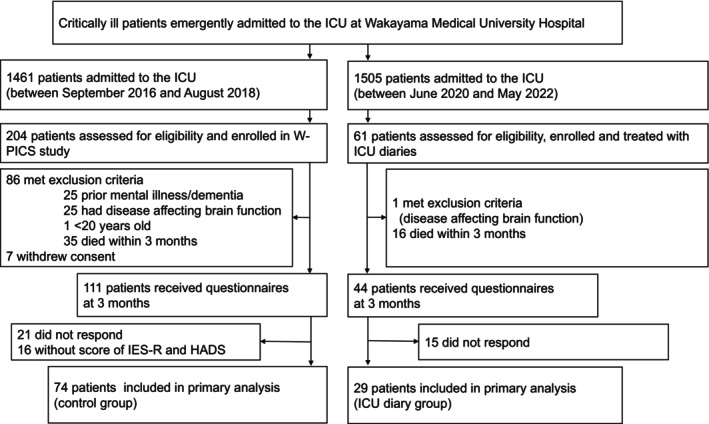
Patient flowchart. The ICU diary group was compared with a control group. The control group was selected from the cohort of the W‐PICS study, which was previously conducted in the same ICU. To select the control group, identical inclusion and exclusion criteria for enrolling patients that received ICU diaries were applied. HADS, hospital anxiety and depression scale; ICU, intensive care units; IES‐R, impact of event scale‐revised; W‐PICS: Wakayama post‐intensive care syndrome.

Enrolled in the study were 61 patients, and they were provided with ICU diaries. One patient was excluded having met the exclusion criteria (a patient with a disease directly affecting brain function). Sixteen patients had died within 3 months, and the remaining 44 patients received questionnaires at 3 months. Finally included in the analysis were the 29 patients among them who responded to the questionnaires in the ICU diary group (Figure 1). Creating a historical cohort from the 204 patients enrolled in Wakayama‐PICS study, patients were selected using identical inclusion and exclusion criteria to those in the present study, and 74 patients were finally included for analysis in the historical cohort (control group) (Figure 1).

Patient characteristics are shown in Table 1. Patients in the ICU diary group were significantly older and had more severe diseases than those in the control group. Regarding other variables, there were no significant differences between the two groups.

**TABLE 1:** Patient characteristics.

Characteristics	Patients without ICU diaries (*n* = 74)	Patients with ICU diaries (*n* = 29)	*p* value
Age, years	68 (59–76)	73 (70–83)	0.008
Male sex	44 (59)	19 (66)	0.66
APACHE II score at ICU admission	18 (15–23)	21 (18–25)	0.021
SOFA score at ICU admission	6 (4–8)	9 (7–11)	0.0012
Charlson comorbidity index	1 (0–2)	1 (0.5–2.5)	0.068
ADL disability (BI ≤ 60) prior to ICU admission^a^	5 (7)	3 (10)	0.68
Admission route			
Emergency department	49 (66)	18 (62)	0.32
Operating room	24 (32)	9 (31)	
General wards	1 (1)	2 (7)	
Reason for ICU admission			
Sepsis	35 (47)	20 (69)	0.21
Trauma	12 (16)	3 (10)	
Cardiovascular	3 (4)	0 (0)	
Others	24 (32)	6 (21)	
Postoperative patients	24 (32)	9 (31)	0.89
Patients received mechanical ventilation during ICU stay	64 (86)	27 (93)	0.50
Ventilator days in ICU, days^b^	4 (2–7)	4 (2–5)	0.50
Tracheostomy in ICU	6 (8)	2 (7)	1.00
Vasopressor therapy during ICU stay, days	2 (0–4)	3 (2–3)	0.58
Kidney replacement therapy during ICU stay	9 (12)	3 (10)	1.00
Patients received corticosteroid during ICU stay^c^	19 (26)	12 (41)	0.15
Length of sedatives or analgesics exposure, days			
Propofol	2 (0–4)	2 (0.5–4)	0.90
Dexmedetomidine	2 (0–4)	2 (0.5–4.5)	0.96
Midazolam	0 (0–0)	0 (0–0)	0.66
Fentanyl	4 (2.8–6)	3 (2–5)	0.43
Delirium in ICU^d^	24 (32)	10 (34)	1.00
Coma in ICU^e^	26 (35)	11 (38)	0.82
Length of ICU stay, days	5 (3–8)	5 (3–6)	0.66
Length of hospital stay, days	32 (19–46)	24 (18–43)	0.45

*Note*: Data are shown as median (interquartile range) or *n* (%).

Abbreviations: APACHE II, acute physiology and chronic health evaluation II; BI, Barthel index; ICU, intensive care unit; SOFA, sequential organ failure assessment.

^a^ Barthel index was missing for 12 patients. Medical records were used in the judgment of whether or not these patients that were missing Barthel Index had ADL disability.

^b^ Ventilator days were calculated after excluding patients who did not receive mechanical ventilation during their ICU stay.

^c^ Patients were considered to have received corticosteroid if the duration of receiving corticosteroid was ≥48 h.

^d^ Delirium was defined as at least one positive C*onfusion Assessment Method for the Intensive Care Unit* through their ICU stay.

^e^ Coma was defined as −4 or −5 of Richmond Agitation and Sedation Scale throughout the day.

In the ICU diary group, 12 out of 29 patients (41%) had read their ICU diaries once or twice by 3 months, and 11 patients (38%) had read them three or more times (Table 2). Nearly 70% patients considered ICU diaries to be either useful or very useful tools. Only two patients (7%) responded that their memories about their ICU stay differed from the contents of their ICU diaries. No patients sought for a referral to the specialized psychiatric outpatient department in our hospital.

**TABLE 2:** How patients treated and interpreted ICU diaries.

	Patients that received ICU diaries (*n* = 29)
How many times patients had read their ICU diaries	
Never	5 (17)
One to two times	12 (41)
Three to five times	9 (31)
Six or more times	2 (7)
Unanswered	1 (3)
To what extent patients felt ICU diaries useful	
Not at all	1 (3)
Not useful	2 (7)
Useful	9 (31)
Very useful	11 (38)
Unanswered	6 (21)
How much difference between patients' memory and ICU diaries	
Very different	2 (7)
A little different	0 (0)
Almost same	10 (34)
Same	4 (14)
Unanswered	13 (45)

*Note*: Data are shown as *n* (%).

Abbreviation: ICU, intensive care unit.

The primary and secondary outcomes are presented in Table 3. In the ICU diary group, four out of 21 patients (19%) experienced PTSD symptoms, compared with 12 out of 73 patients (16%) in the control group (*p* = 0.75). The median (interquartile range [IQR]) IES‐R scores were 4 (2–18) and 9 (3–19) in ICU diary and control groups, respectively (*p* = 0.30). In the multivariate logistic regression analysis adjusted for predefined confounders, no significant associations were found between the use of ICU diaries and the presence of PTSD symptoms at 3 months after ICU discharge (crude odds ratio [OR] 1.20: 95% confidence interval [CI] 0.34–4.19: *p* = 0.78; adjusted OR 0.98: 95% CI 0.26–3.70: *p* = 0.98). As sensitivity analysis, similar results were obtained from propensity score‐stratifying analysis (adjusted OR 1.06: 95% CI 0.25–4.53).

**TABLE 3:** Primary and secondary outcomes.

	Patients that did not receive ICU diaries (*n* = 74)	Patients that received ICU diaries (*n* = 29)	*p* value
IES‐R			
Data available	73 (99)	21 (72)	
Score	9 (3–19)	4 (2–18)	0.30
Score ≥ 25	12 (16)	4 (19)	0.75
HADS‐anxiety			
Data available	74 (100)	24 (83)	
Score	6 (2–9)	4 (2–7.75)	0.45
Score ≥ 8	28 (38)	6 (25)	0.33
HADS‐depression			
Data available	73 (99)	24 (83)	
Score	6 (4–10)	6.5 (3–9.75)	0.51
Score ≥ 8	33 (45)	7 (29)	0.23
Barthel index			
Data available	74 (100)	27 (93)	
Score	100 (75–100)	100 (70–100)	0.76
Score ≤ 60	14 (19)	6 (22)	0.78

*Note*: Data are shown as median (interquartile range) or *n* (%).

Abbreviations: HADS, hospital anxiety and depression scale; IES‐R, impact of events scale‐revised.

Regarding anxiety and depression symptoms, there was a numerically lower prevalence of these symptoms in the ICU diary group compared with in the control group, although there was no statistically significant association between ICU diaries and these symptoms (anxiety symptoms: crude OR 0.54: 95% CI 0.19–1.54: *p* = 0.25; adjusted OR 0.46: 95% CI 0.15–1.38: *p* = 0.17; depression symptoms: crude OR 0.50: 95% CI 0.18–1.35: *p* = 0.17; adjusted OR 0.40: 95% CI 0.14–1.16: *p* = 0.090).

## DISCUSSION

The use of ICU diaries in critically ill patients was not associated with a reduced incidence of PTSD symptoms at 3 months when compared with the historical cohort. Additionally, a numerically lower incidence of depression and anxiety symptoms was noted in patients treated with ICU diaries compared with those without ICU diaries; however, the difference did not reach statistical significance. Approximately 90% of patients found ICU diaries useful, and only two patients (7%) reported differences between their own memories and the content of the ICU diaries.

Regarding PTSD symptoms, a previous study reported that a patient's delusional memories without factual memories of their ICU stay were associated with PTSD symptoms after ICU discharge.^5^ Theoretically, ICU diaries aim to reduce PTSD symptoms by replacing delusional memories with factual ones. A before‐after comparison study conducted in Japan demonstrated that the implementation of ICU diaries was associated with a reduced intensity of anxiety, depression, and PTSD symptoms among patients with distorted memories of their ICU stay.^21^ However, only a small percentage of patients in the current study reported differences between their memories and the contents recorded in ICU diaries. This might explain why an association between ICU diaries and the improvement of PTSD symptoms could not be identified.

There are conflicting results regarding the impact of ICU diaries on psychiatric symptoms, including PTSD symptoms, depression, and anxiety. Several small studies showed a reduction in psychiatric symptoms when ICU diaries were paired with debrief sessions, where healthcare professionals read the diaries with the patients and ensured understanding.^22^, ^23^, ^24^ By contrast, a multicenter randomized trial involving 657 critically ill patients which examined the effect of ICU diaries without post‐discharge debrief reported similar prevalence of PTSD symptoms between patients treated with and without ICU diaries.^9^ The manner of implementing ICU diaries, such as incorporating post‐discharge debrief sessions, might influence their effectiveness. In the current study, efforts were made to conduct debrief sessions led by ICU physicians when handing the ICU diaries to the patients. However, debrief sessions could not be performed for all patients, potentially influencing the effectiveness of ICU diaries in this study.

Previous studies reported that many critically ill patients experienced long‐lasting psychiatric symptoms over a year, significantly impacting their quality of life.^4^, ^25^, ^26^ However, at the time of writing, there were no established preventive measures for these psychiatric symptoms. The current results did not reach statistical significance, but they did detect a potential signal indicating the effectiveness of ICU diaries in preventing depression and anxiety associated with PICS, warranting further exploration in future studies.

This study had several limitations. Firstly, it was a single‐arm intervention study using a historical cohort as a control. Consequently, there were between‐group differences, such as the older age and increased severity of patients in the ICU diary group, which might influence the study results. However, potential selection bias was mitigated by employing identical inclusion and exclusion criteria in the same ICU for both the ICU diary group and the control group. Furthermore, throughout the study period, which included the Wakayama‐PICS study, there were no major advancements in the prevention and treatment of psychiatric symptoms in PICS. In fact, there was no change in our institute's prevention and treatment strategies for psychiatric symptoms in PICS except for family visitation policy. From September 2020 to the end of this study (almost the entire period of ICU diary group), restrictions on family visits were introduced in our institute due to coronavirus disease 2019 pandemic. Family visitation restrictions were previously reported to be associated with an increased prevalence of post‐discharge psychiatric disorder.^27^ ICU diaries, which were almost simultaneously introduced with family visitation restriction, were not associated with increased prevalence of psychiatric symptoms in our study. This reinforces rather than weakens the hypothesis that ICU diaries have some favorable effect on psychiatric symptoms. Additionally, baseline characteristics were carefully adjusted for, including age and severity, using predefined adjusters to evaluate outcomes.

Secondly, the evaluation in this study focused on patients' psychiatric symptoms, and symptoms in their family members were not assessed. ICU diaries may be a useful intervention for the patients' family members.^28^ Unfortunately, there was a lack of information about the potential effects of ICU diaries on the psychiatric symptoms of patients' family members.

Thirdly, this was a single‐center study, and as such, the generalizability of the results might be limited. Furthermore, this study was descriptive and exploratory research, the main purpose of which was to describe and explore the effect of ICU diaries, not to be a confirmatory study, and sample sizes were not calculated to confirm the effect of the ICU diaries. The results of this study should therefore be considered as hypothesis‐generating. To gain a more precise understanding of the effect of ICU diaries on psychiatric symptoms, a multicenter study on ICU diaries is warranted. Moreover, it will be particular important to select high‐risk patients to evaluate the effect of ICU diaries on psychiatric symptoms.

## CONCLUSIONS

The intervention of ICU diaries among critically ill patients was not associated with a reduced incidence of PTSD symptoms at 3 months after ICU discharge. While a numerically lower prevalence of anxiety and depression symptoms was observed in patients treated with ICU diaries compared with the control group, this difference did not reach statistical significance.

## CONFLICT OF INTEREST STATEMENT

Dr. Miyamoto has received lecturer fees from Asahi Kasei Pharma, Japan Blood Products Organization, and Chugai Pharmaceutical.

## ETHICS STATEMENT

Approval of the research protocol: The study was approved by the Wakayama Medical University Ethics Boards (approval number 2884).

Informed consent: Written informed consent was obtained from patients or their family members for the study participation and publication.

Registry and the registration no. of the study/trial: This study was retrospectively registered to the UMIN Clinical Trial Registry on 9th November, 2020 after the initiation of patient enrollment, given the noninvasive nature of our study (registration no. UMIN000042310, https://center6.umin.ac.jp/cgi‐open‐bin/ctr_e/ctr_view.cgi?recptno=R000048303).

Animal studies: N/A.

## Supporting information


Figure S1.

## Data Availability

The data that support the findings of this study are available from the corresponding author upon reasonable request.
