# Supplementary material for: The effect of ICU diary on psychiatric symptoms after ICU discharge among adult critically ill patients: A prospective comparative study
Source: Acute Med Surg. 2024 Nov 28;11(1):e70026. doi: 10.1002/ams2.70026 (PMC11603429; doi:10.1002/ams2.70026)
Supplement: Supplementary file 1 — Figure S1. [file AMS2-11-e70026-s001.pdf]

ICU日記

The ICU diary

Patient's name

お名前

年　　月　　日

Period of ICU stay
DD/MM/YY to DD/MM/YY

～　　年　　月　　日

Wakayama Medical University Hospital
Intensive Care Unit

和歌山県立医科大学附属病院
集中治療室（ICU）

あなたのご病気は…

Your illness was...

You were admitted to the ICU in DD/MM/YY
We are committed to supporting your recovery with all our strength.

年　月　日 集中治療室に入院されました

あなたが一日でも早く良くなるように私達は全力でサポートします

月　　日（　）天気

DD/MM/YY (day of the week), Weather

医師より

Posts from your physicians

リハビリの先生より

Posts from your physiotherapists

看護師より

Posts from your nurses

月　　日（　）天気

DD/MM/YY (day of the week), Weather

写真

Your photos on this day

看護師より

Posts from your nurses

リハビリの先生より

Posts from your physiotherapists

医師より

Posts from your physician

！！注意！！

・できればご家族の方とお読みになってください。

・この日記をご覧になって集中治療室での記憶がよみがえり、非常に強いストレスをお感じになられた場合はそれ以上読まないでください。

・ストレスによる強い症状を自覚されている場合は医師の診察を受けることができますので、下記の連絡先へご相談ください。

・その他、日記に関してご不明な点がある場合も下記にご連絡ください。

!!Caution!!

We recommend that, if possible, you read this diary with your family members.

This ICU diary may help you recall memories from your ICU stay. However, if reading it causes you emotional distress, we suggest that you stop reading.

If you experience any symptoms related to stressful memories from your ICU stay, please feel free to consult with our physicians.

If you have any questions, don't hesitate to contact us using the details below.

和歌山県立医科大学　救急・集中治療医学講座
〒XXX-XXXX
Tel: XXX-XXX-XXXX
Fax: XXX-XXX-XXXX
E-mail: XXXX@XXXX.XX.XX

Department, Institute
Address
Phone number
FAX number
Mail address
